# Systematic Study of the SiO_x_ Film with Different Stoichiometry by Plasma-Enhanced Atomic Layer Deposition and Its Application in SiO_x_/SiO_2_ Super-Lattice

**DOI:** 10.3390/nano9010055

**Published:** 2019-01-03

**Authors:** Hong-Ping Ma, Jia-He Yang, Jian-Guo Yang, Li-Yuan Zhu, Wei Huang, Guang-Jie Yuan, Ji-Jun Feng, Tien-Chien Jen, Hong-Liang Lu

**Affiliations:** 1 State Key Laboratory of ASIC and System, Shanghai Institute of Intelligent Electronics & Systems, School of Microelectronics, Fudan University, Shanghai 200433, China; hpma@fudan.edu.cn (H.-P.M.); 18212020044@fudan.edu.cn (J.-H.Y.); jianguoyang13@fudan.edu.cn (J.-G.Y.); 18212020054@fudan.edu.cn (L.-Y.Z.); eehuangw@fudan.edu.cn (W.H.); 2 SMIT Center, School of Automation and Mechanical Engineering, Shanghai University, Shanghai 201800, China; guangjie@shu.edu.cn; 3 Shanghai Key Laboratory of Modern Optical System, School of Optical-Electrical and Computer Engineering, University of Shanghai for Science and Technology, Shanghai 200093, China; fjijun@usst.edu.cn; 4 Department of Mechanical Engineering Science, University of Johannesburg, Johannesburg ZA-2006, South Africa; tjen@uj.ac.za

**Keywords:** SiO_x_, SiO_2_, plasma-enhanced atomic layer deposition (PEALD), stoichiometry, superlattice

## Abstract

Atomic scale control of the thickness of thin film makes atomic layer deposition highly advantageous in the preparation of high quality super-lattices. However, precisely controlling the film chemical stoichiometry is very challenging. In this study, we deposited SiO_x_ film with different stoichiometry by plasma enhanced atomic layer deposition. After reviewing various deposition parameters like temperature, precursor pulse time, and gas flow, the silicon dioxides of stoichiometric (SiO_2_) and non-stoichiometric (SiO_1.8_ and SiO_1.6_) were successfully fabricated. X-ray photo-electron spectroscopy was first employed to analyze the element content and chemical bonding energy of these films. Then the morphology, structure, composition, and optical characteristics of SiO_x_ film were systematically studied through atomic force microscope, transmission electron microscopy, X-ray reflection, and spectroscopic ellipsometry. The experimental results indicate that both the mass density and refractive index of SiO_1.8_ and SiO_1.6_ are less than SiO_2_ film. The energy band-gap is approved by spectroscopic ellipsometry data and X-ray photo-electron spectroscopy O 1s analysis. The results demonstrate that the energy band-gap decreases as the oxygen concentration decreases in SiO_x_ film. After we obtained the Si-rich silicon oxide film deposition, the SiO_1.6_/SiO_2_ super-lattices was fabricated and its photoluminescence (PL) property was characterized by PL spectra. The weak PL intensity gives us greater awareness that more research is needed in order to decrease the x of SiO_x_ film to a larger extent through further optimizing plasma-enhanced atomic layer deposition processes, and hence improve the photoluminescence properties of SiO_x_/SiO_2_ super-lattices.

## 1. Introduction

Compatible with common micro-electronic device fabrication techniques and materials [1,2], silicon (Si) based micro-nano devices have become the most promising material for advanced integrated opto-electronic technologies in the future [3]. Since the discovery of efficient photoluminescence in the red region in porous Si at room temperature [4], silicon nanocrystals (Si-NCs) were extensively studied in the last decade [5,6]. Particularly in recent years, Si-NCs have aroused significant attention in the applications of light sources and “all-silicon” devices like silicon lasers, light emitting diode (LED), flash memories [7,8], and tandem solar cells. Among the different techniques of Si-NCs fabrication in a solid matrix, super-lattices were considered to be very effective in producing size-, density-, and shape-controlled Si-NCs [9,10,11].

A large number of studies have been reported on Si-NCs embedded in silicon dioxide (SiO_2_) [12,13,14]. Based on the quantum confinement theory [15,16,17], the correlation between the photoluminescence (PL) properties and the size of the nanocrystals has been established. Control over all three parameters (size, density, spherical shape) can be reached by depositing thin alternating layers of stoichiometric and Si rich dielectrics in the form of a superlattice (SL). This approach has been well established for silicon nanocrystals in SiO_2_ matrix. However, most of the Si-NCs and superlattices in previous studies are fabricated by chemical vapor deposition (CVD) [18,19,20], sputtering [21,22,23], or other deposition techniques [24], these techniques are good at depositing thick film but weak in thin film. Which hamper the further study on the control of Si-NCs in superlattice with ultrathin dielectric film or barrier layer. Atomic layer deposition (ALD) is a promising technology for advanced thin film deposition as it offers excellent control at the atomic scale over the thickness and uniformity of the film [25,26,27]. It allows the precise preparation of size- and distribution-controlled silicon nanocrystals. So, it will be a wonderful opportunity to fabricate silicon oxides and related superlattice by ALD technique, and study its photoluminescence properties.

There have been a certain amount of studies on the growth of SiO_2_ film using ALD [28,29,30], but rarely on the control of its stoichiometry and optical/electrical properties. Thus, in this study, plasma- enhanced atomic layer deposition (PEALD) was used to deposit SiO_x_ film with different stoichiometry. In detail, ALD deposition parameters were firstly studied to understand the effect of deposition parameters like precursor pulse time and temperature on the growth properties of SiO_x_ film. Upon choosing suitable deposition parameters, three kinds of film (SiO_2_, SiO_1.8_, and SiO_1.6_) with different stoichiometry were acquired successfully. Film properties like physical, chemical, and photo-electrical properties of SiO_x_ film with different stoichiometry were studied systematically. Specifically, basic film characteristics including microstructure, density, and roughness were evaluated, while the chemical bonding character of the obtained film was discussed by Si 2p and O 1s in detail. Furthermore, the energy band-gap of the SiO_x_ film with different stoichiometry was identified by both spectroscopic ellipsometry (SE) data analysis and X-ray photo-electron spectroscopy (XPS) measurements. Finally, to examine the properties of Si rich SiO_x_ obtained by ALD in this study, SiO_1.6_/SiO_2_ superlattice was fabricated and its photoluminescence property was characterized by PL spectra.

## 2. Experimental Section

### 2.1. Film Preparation

**Figure 1:**
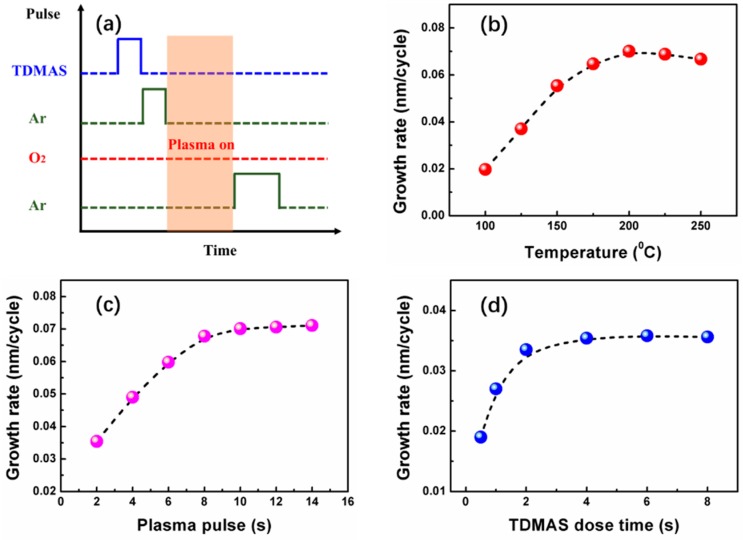
(**a**) Parameters of one atomic layer deposition (ALD) cycle of the SiO_x_ growth utilized in this work. Growth rate of SiO_x_ films in dependence on (**b**) temperature, (**c**) plasma pulse time (with 2 s tris(dimethylamino)silane (TDMAS) pulse time), and (**d**) TDMAS pulse time (with 2 s plasma pulse time).

SiO_x_ films were deposited on p-type (1–10 Ω·cm), single polished, Si(100) wafers in a BENEQ TFS200 ALD system (BENEQ, Finland) at a vacuum degree of 1 mbar. Prior to deposition, the Si wafers were cleaned by a standard RCA process followed by a deionized water rinsing and drying in N_2_. During the deposition process, precursors of Si and O were tris(dimethylamino)silane (TDMAS) and O_2_ plasma. TDMAS was purchased from Fornano company and maintained at 20 °C in a stainless bottle. The O_2_ plasma was activated at 200 W. The schematic diagram of one ALD cycle of SiO_x_ growth utilized in this work is illustrated in Figure 1a. Each ALD cycle contains four steps: TDMAS pulse, Ar purge, plasma processing, and Ar purge.

### 2.2. Sample Characterization

The surface morphology was observed using an atomic force microscope (AFM, Bruker, icon), and a typical 5 μm × 5 μm area was investigated using non-contact mode. Transmission electron microscopy (TEM) (FEI, TECNAI G2 F20) was used to analyze the micro-structure and interface composition of the silicon oxides. The chemical bonding character of the obtained film was characterized by XPS (SPECS, Berlin, Germany) using a mono-chromatic Al Kα source (hν = 1486.6 eV). A narrow scan resolution of 0.1 eV was used. The adventitious C 1s peak, arising from traces of hydrocarbon in the spectrometer, was used as a reference for evaluating the peak positions because of static charging of samples. The C 1s peak position was observed together with other peaks (Si 2p, N 1s, and O 1s) of the spectrum, and all the XPS spectra were calibrated by the C 1s peak at a binding energy of 284.6 eV. The micro-structure and morphology of the film were characterized by X-ray reflection (XRR) (Bruker, D8, Billerica, MA, USA). SE measurements were carried out on a rotating analyzer ellipsometer (SOPRA, GES-5E, Annecy, France). The incident angle was 75°. The spectral wavelength range from 190 to 800 nm, the system measured the spectra of Ψ and Δ as functions of wavelength (λ). The resulting spectra was fitted with WinElli_II software. PL spectroscopy was used to investigate the optical properties of Si-NCs produced within the SiO_x_/SiO_2_ super-lattice. The PL was excited by the 325 nm line of HeCd laser. The PL signal was focused into a single monochromator and detected by a nitrogen-cooled charge-coupled device (CCD) camera. All spectra were corrected for the spectral response of the measurement system.

## 3. Results and Discussion

### 3.1. Film Fabrication and Growth Rate Experiments

In order to optimize the growth parameters needed for the self-limiting deposition of SiO_x_ thin film, the effect of temperature, TDMAS dose, and O_2_ plasma duration was studied. At first, with the purpose of studying the effect of temperature on growth rate, 100 cycles with 2 s TDMAS and 8 s O_2_ plasma was deposited at different temperatures (100–250 °C). The growth rate increased initially as the temperature was increased from 100 to 175 °C. However, it stayed nearly constant at an ALD temperature window from 175 °C to 250 °C, as seen in Figure 1b, where deposition rate was constant at 0.07 nm/cycle. Figure 1c,d display the growth rate of the SiO_x_ thin film as a function of plasma time and TDMAS pulse time at a deposition temperature of 200 °C. The O_2_ flow rate was fixed at 50 sccm. The growth rate was determined here in terms of the film thickness divided by the total number of applied ALD cycles. The film thickness was 7.08, 9.8, 11.96, 13.56, 14.02, 14.13, and 14.21 nm for the samples with the plasma pulse time of 2, 4, 6, 8, 10, 12, and 14 s, respectively. The total ALD cycles were kept at 200 for the simulation. It can be observed from Figure 1c that the growth rate increases initially as plasma time increases. It remained constant at 0.071 nm/cycle when the plasma time was greater than 8 s. To obtain the saturated TDMAS pulse time, the plasma pulse time was kept at 8 s. The film thickness became 3.8, 5.4, 6.7, 7.08, 7.16, and 7.12 nm when the TDMAS pulse time was set as 0.5, 1, 2, 4, 6, and 8 s, respectively. As seen in Figure 1d, deposition rate was saturated for TDMAS pulse time starting from 2 s. These results suggest that the SiO_x_ thin film was grown in a self-limiting manner when using the PEALD technique.

**Figure 2:**
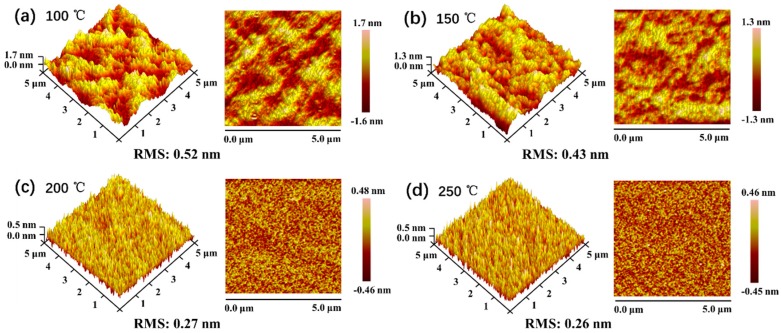
Three-dimensional and two-dimensional AFM images of SiO_2_ films deposited at (**a**) 100 °C, (**b**) 150 °C, (**c**) 200 °C, and (**d**) 250 °C, respectively.

Figure 2 shows the 5 μm^2^ × 5 μm^2^ AFM images for SiO_2_ thin films with different deposition temperatures. It is clear that the morphology changes with different temperatures. As the deposition temperature increases, the resulting morphology transforms from an island structure into a slim needle like structure. The surface root mean-square (RMS) roughness becomes lower with the increase in deposition temperature. For SiO_2_ thin film deposited at 100 °C, it presents the maximum roughness of 0.52 nm, then the value decreases to 0.43, 0.27, and 0.26 nm for the films deposited at 150, 200, and 250 °C, respectively. It was found that the surface roughness of all the films prepared in this work is very small, indicating that the films have a smooth surface.

Then, the three sample sets (S1, S2, and S3) were prepared in order to obtain SiO_x_ film with different stoichiometry. In ALD deposition, the temperature was fixed at 250 °C. The precursor pulse time was changed in order to obtain silicon oxides of stoichiometric (SiO_2_) and non-stoichiometric (SiO_x_, x < 2). To be specific, TDMAS pulse time was 2, 2, and 4 s for S1, S2, and S3 sample, respectively, O_2_ plasma duration was 8, 4, and 2 s for S1, S2, and S3 film, respectively. The purge time was always kept at 4 s no matter whether it was after the TDMAS pulse or after the O_2_ plasma. Meanwhile, in order to decrease the oxygen content in the film to form Si rich silicon dioxide, the O_2_ flow rate was also adjusted, it was 50 sccm, 50 sccm, and 25 sccm on the deposition of S1, S2, and S3 film, respectively.

**Figure 3:**
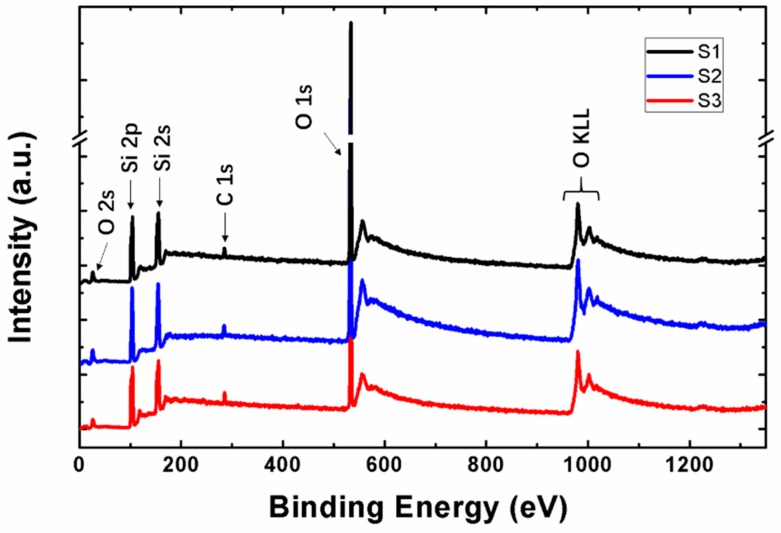
X-ray photo-electron spectroscopy (XPS) survey scans of SiO_x_ film with different stoichiometry.

After deposition, all samples were tested by XPS to determine the stoichiometric. Figure 3 displays the XPS wide scan spectrum of S1, S2, and S3 film after Ar^+^ ion treatment. The determined XPS peaks of Si, O, and C are stated in the figure. All the peaks of elements are marked and recognized in detail. By comparing the content of Si and O element in three samples, it is found that the stoichiometries are x = 2, 1.8, and 1.6 for S1, S2, and S3 film, respectively. Which means that the S1 sample is the silicon dioxide film of stoichiometric and S2 and S3 samples are silicon dioxide of nonstoichiometric.

### 3.2. Properties of SiO_x_ Film with Different Stoichiometry

**Figure 4:**
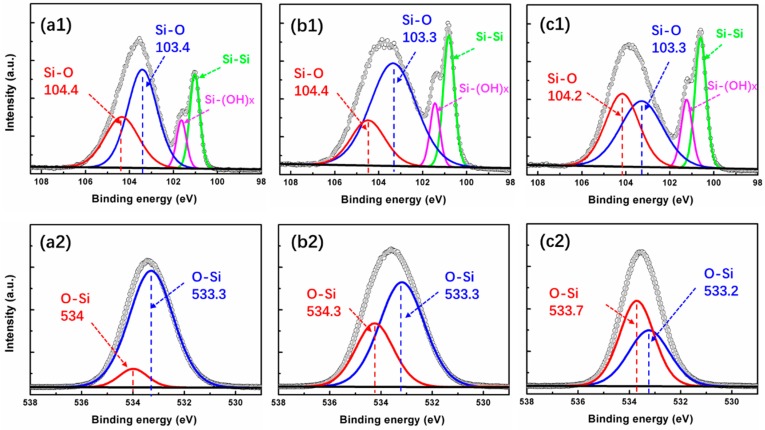
XPS analyses of SiO_2_ (**a1**,**a2**), SiO_1.8_ (**b1**,**b2**), and SiO_1.6_ (**c1**,**c2**) samples.

To further analyze the concentration, microstructure, chemical valence, and composition of the film and elements, the Si 2p and O 1s spectra of the SiO_x_ sample were examined by high-resolution XPS, as shown in Figure 4. From the Si 2p spectra of the S1 film (Figure 4a1), four peaks are observed at 104.4, 103.4, 101.6, and 101 eV, corresponding to the Si-O (SiO_2_), Si-O (SiO_2_), Si-OH_x_ (Si(OH)_x_), and Si-Si (Si substrate) bonds [31], respectively. For the S2 sample, four sub-peaks centered at 104.4, 103.3, 101.4, and 100.8 eV are observed. They are assigned to be the Si-O (SiO_2_), Si-O (SiO_2_), Si-OH_x_ (Si(OH)_x_), and Si-Si (Si substrate) bonds, respectively. It is worth pointing out that the intensity and the area of the Si-OH_x_ and Si-Si related sub-peaks increased compared to the strength in S1 sample, which means more content of silicon-related clusters became residual in the film. For the S3 sample, the four sub-peaks are centered at 104.2, 103.3, 101.2, and 100.6 eV. The intensity and area of the Si-OH_x_ related sub-peak further increased, which is induced by the decrease in O_2_ plasma duration and flow rate. By comparing the O 1s spectra of S1 (Figure 4a2), S2 (Figure 4b2) and S3 sample (Figure 4c2), it can be found that the O 1s spectra in all three films can be fitted mainly by two sub-peaks located at ~534 eV and ~533.3 eV. For S1, it is 534 eV and 533.3 eV, for S2, it is 534.3 eV and 533.3 eV, then for S3, it is 533.7 eV and 533.2 eV. When we compare these two sub-peaks with the sub-peaks related to Si-O bonds in Si 2p peak (for a better view, they were both marked as the same red and blue color, respectively), we find that they present the same variation tendency in these three samples. Evidently, in Si 2p peaks, the sub-peak related to Si-O bonds with higher binding energy (red line) changed from 104.4 eV to 104.2 eV when reducing the oxygen content in SiO_x_ film. As a matter of fact, the sub-peak related to O-Si bonds with higher binding energy (red line) also shifted from 534 eV to 533.7 eV. Therefore, it is supposed that the two sub-peaks related to O-Si bonds in the O 1s peak (Figure 4a2–c2) correspond with the two sub-peaks related to Si-O bonds of the matching sample found in Si 2p (Figure 4a1–c1). Moreover, the distance of the two sub-peaks related to Si-O bonds decreased from 1.0 eV to 0.9 eV, while, the value of the two sub-peaks related to O-Si bonds decreased from 0.7 to 0.5 eV as well. In addition to these variations, the relative intensity of these two sub-peaks corresponding to high and low binding energy also had the same variation trend both in Si 2p and O 1s. All these results indicate that the chemical binding energy and microstructure are different in these SiO_x_ film with different stoichiometry.

**Figure 5:**
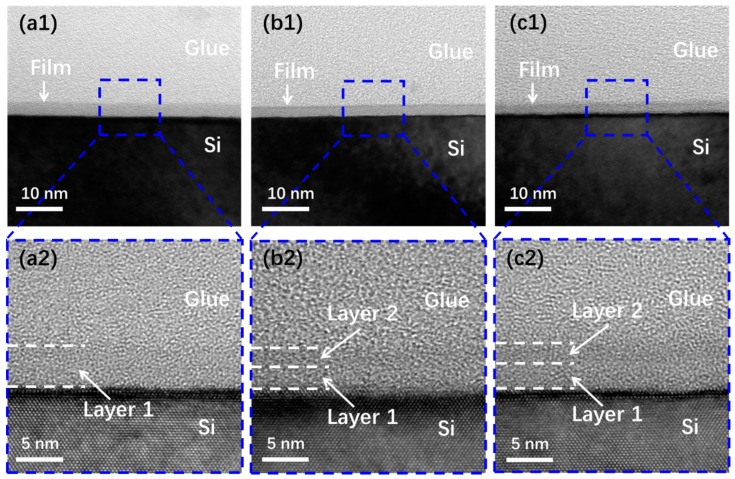
TEM images of SiO_2_ (**a1**,**a2**), SiO_1.8_ (**b1**,**b2**), and SiO_1.6_ (**c1**,**c2**) samples on Si substrate.

For further analysis, the TEM test was used to verify the microstructure like surface roughness and the interfacial composition of these three films. The surface morphology of the three samples can be observed by the low-resolution TEM images shown in Figure 5a1–c1. It can be seen that all films present a smooth surface. This finding agrees with the surface morphology and roughness data shown in Figure 2c, since all films were deposited at 200 °C. The high-resolution TEM images shown in Figure 5a2–c2 exhibit different film structure. As shown in Figure 5a2, the S1 sample is just composed of one layer, which is obviously the SiO_2_ film. For S2 sample, two sublayers are detectable in Figure 5b2, layer 1 is assigned to the SiO_2_ owing to the native oxidation on the substrate surface. It also comes from the contribution of residual oxygen in the reactor chamber. It should be noted that residual oxygen is unavoidable since the vacuum is just about 1 mbar. Therefore, at the beginning of the ALD process, the oxygen flow was unstable and not the value that we desired. As a result, SiO_2_ was generated more or less on the surface of the Si substrate. After few ALD cycles, the residual oxygen in the chamber ran out. Then, silicon oxides with the desired stoichiometry could be obtained when the oxygen flow began to stabilize. Based on this, layer 2 in Figure 5b2 is assigned to the SiO_1.8_ layer. Moreover, for the S3 sample shown in Figure 5c2, a bilayers structure can also be found. Same as the S2 sample, the layers 1 and 2 are assigned to SiO_2_ and SiO_1.6_, respectively. This finding is in agreement with the variation of the Si-O bonds in the Si 2p spectra and O-Si bonds in the O 1s spectra. To be specific, as seen in Figure 4, both of Si-O and O-Si bonds shift to lower binding energy with the Si/O ratio changing from 2 to 1.6. According to reports in previous research, higher valence of Si ion corresponds to higher binding energy [32,33]. On the basis of this finding, there is reason to believe that a certain amount of Si ion turns to lower valence in S2 and S3 sample. This can be verified both by the Si 2p spectra in Figure 4b1–c1 and the TEM structure in Figure 5b2–c2. Meanwhile, because of the existence of more valence states in S2 and S3 sample, the Si 2p and O 1s spectra in these two samples present broaden profile than the value in S1 sample, as seen in Figure 4.

**Figure 6:**
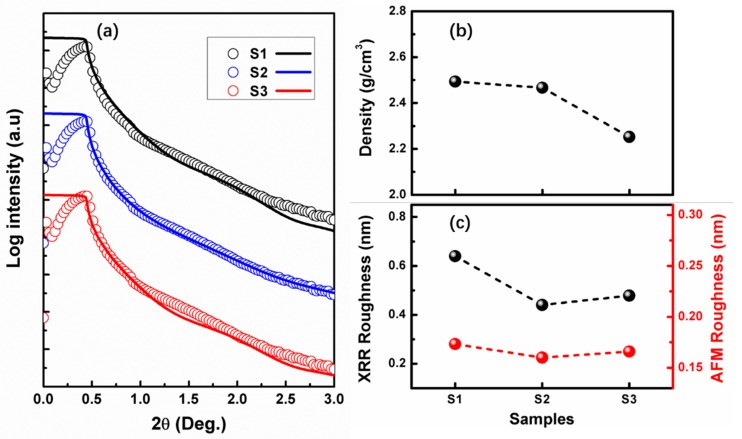
(**a**) Measured and simulated X-ray reflection (XRR) curves of three kinds of SiOx film. (**b**) Density, and (**c**) root mean-square (RMS) roughness of these films obtained by XRR simulation.

Figure 6a shows the measured (scatter line) and simulated (solid line) XRR curve of the SiO_x_ film with different stoichiometry. Figure 6b,c shows the film density and roughness obtained by XRR simulation. The density of SiO_2_ (S1) sample was ~2.49 g/cm^3^, and it became 2.46 g/cm^3^ for the SiO_1.8_ (S2) film. Then, the value decreased to 2.25 g/cm^3^ for SiO_1.6_ (S2) film. This result indicated that the mass density of silicon dioxide decreases with the decrease of oxygen concentration, in other words, the film mass density becomes lower for Si rich silicon dioxide (SiO_1.6_) than SiO_2_. Meanwhile, surface roughness obtained by XRR and AFM is also shown in Figure 6c. It was found that the surface roughness of all the films prepared in this work is very small, which means that the film is very smooth. It should be noted here that both XRR and AFM data present a similar tendency of variation for roughness. Obviously, the RMS result simulated from XRR data is higher than the value obtained by AFM, which is in agreement with the result reported in other work [34]. As seen from RMS values obtained by XRR, the SiO_2_ film exhibits the highest RMS roughness value of about 0.64 nm, and the value of the other film is around 0.4–0.5 nm.

**Figure 7:**
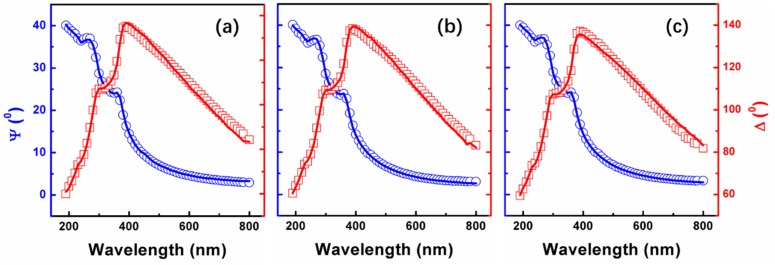
Ellipsometry measured and simulated Ψ (blue line) and Δ (red line) for (**a**) S1(SiO_2_), (**b**) S2(SiO_1.8_), and (**c**) S3(SiO_1.6_) samples.

**Figure 8:**
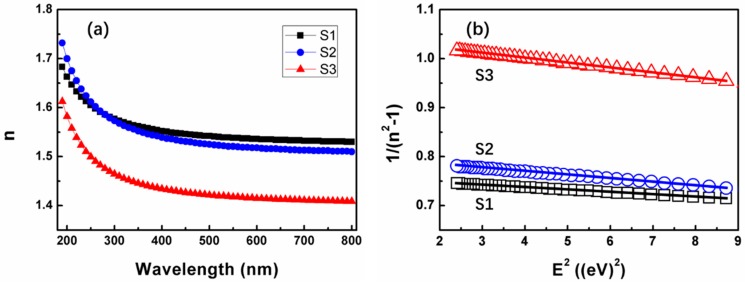
(**a**) Refractive index and of SiOx thin films as a function of the wavelength, (**b**) dependence of 1/(*n*^2^ − 1) as a function of square photon energy obtained from SE (symbols) and linear fit of this data (solid lines) according to Wemple-DiDomenico model [36].

In order to achieve in-depth knowledge on the evolution of the optical properties of SiO_x_ film with different stoichiometry, SE measurements have been performed at room temperature immediately after removing the samples from the deposition system to avoid surface contamination. The measured (scatter line) and simulated (solid line) Ψ and Δ of SiO_x_ thin films for angles of incidence 75° are shown in Figure 7. From this data, the refractive index of the SiO_x_ can be deduced through a fitting-based analysis. Because of the excellent transparency of the SiO_x_ in the considered wavelength range, it is valid to use a Cauchy relation to model the refractive index of the SiO_x_: $n\left(\lambda \right)=A+B/\lambda^{2}$ + $C/\lambda^{4}$. As can be seen in Figure 7, an excellent fit (solid lines) is achieved. Then, the optical constants of SiO_x_ thin film is extracted and obtained from the fitted Ψ and Δ. Figure 8a shows the changes of the refractive index (n) as a function of wavelength in the range of 190–800 nm. Evidently, this fitting procedure yielded a refractive index value (n) of 1.54 at a wavelength of 632.8 nm for SiO_2_, in agreement with the value reported in the literature [35]. Then for SiO_1.8_ and SiO_1.6_, it becomes 1.51 and 1.42, respectively. Therefore, the refractive index of the SiO_x_ film can be affected by varying the oxygen concentration.

**Figure 9:**
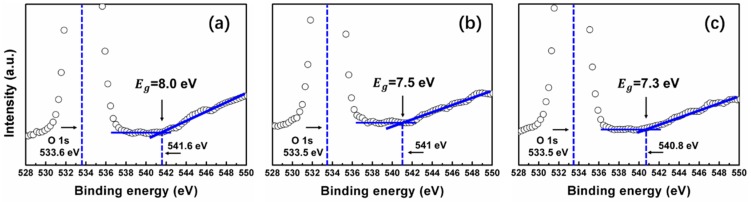
The determination of *E_g_* through the O 1s peak analysis through XPS measurement for samples (a) SiO_2_, (b) SiO_1.8_, and (c) SiO_1.6_.

The refractive index dispersion *n* of SiO_x_ film was further fitted by the Wemple-DiDomenico model [36] with the purpose of determining the oscillator energy and strength. The refractive index data can be fitted in the spectra below the band-gap to the single oscillator expression [37]:

(1) $$n^{2}=1+\frac{E_{0}\cdot E_{d}}{E_{0}^{2}-E^{2}}$$

where *E* represents the photon energy, *E*_0_ measures oscillator energy, and *E_d_* represents dispersion energy, which measures the oscillator strength (the strength of inter-band optical transitions). From linear regression of dependence (*n*^2^ − 1)^−1^ against *E*^2^ (as shown in Figure 8b), the parameters *E*_0_ and *E_d_* can be calculated. The values of the fitting constants *E*_0_ and *E_d_* are given in Table 1. We can see that for SiO_2_ sample, *E*_0_ is ~12.5 eV, *E_d_* is ~16.5 eV. For SiO_1.8_ sample, *E*_0_ is ~10.5 eV, *E_d_* is ~12.8 eV. Then, for SiO_1.6_ sample, *E*_0_ is ~10.4 eV, *E_d_* is ~9.8 eV. The oscillator energy, *E*_0_, is an ‘average’ energy gap, as pointed out in many references [38,39,40]. S.H. Wemple et al. [36] found out that *E*_0_ is related empirically to the lowest direct energy band-gap *E_g_* by *E*_0_ ≈ 1.5 *E_g_*. The values of *E_g_* determined in this way are 8.3, 7.3, and 6.9 eV for SiO_2_, SiO_1.8_, and SiO_1.6_, respectively. Meanwhile, *E_g_* of these SiO_x_ film was also determined by XPS O 1s data, which has been reported by many scientists [41,42,43]. As shown in Figure 9, a representative high-resolution scan of the O 1s core level of SiO_2_, SiO_1.8_, and SiO_1.6_ film are used to determine the energy band-gap of these three films. We can notice that the *E_g_* is 8, 7.5, and 7.3 eV for SiO_2_, SiO_1.8_, and SiO_1.6_ film, respectively. As shown in Table 1, the energy band-gap obtained by O 1s peak is very close to the value obtained by the *E*_0_ simulation above. This result confirms that the energy band-gap of SiO_x_ films decrease with decreasing oxygen composition.

For the dispersion energy an empirical relation is established [36]: *E_d_* = *βN*_c_*N*_e_*Z*_a_, where *β* is a constant, according to Wemple, *β* has a value of 0.37~0.04 eV. *N*_c_ is the coordination number of the nearest neighboring cation to the anion, and *Z*_a_ is the formal chemical valency of the anion, then *N*_e_ is the total number of valence electrons per anion [39]. For SiO_2_, *N*_e_ = [(4 valence electrons)·(1 silicon cation) + (6 valence electrons)·(2 oxygen anions)]/2 = 8, and *Z*_a_ = 2. For SiO, *N*_e_ = [(4 valence electrons)·(1 silicon cation) + (6 valence electrons)·(1 oxygen anions)]/1 = 10, and *Z*_a_ = 2. Thus, *N*_c_ can be calculated from the relationship: *N*_c_ = *E_d_*/*βN*_e_ *Z*_a_. It is noted that the values of *E_d_* and *N*_c_ decreases with the decrease of oxygen concentration in SiO_x_ film. So, we can conclude that SiO_x_ film has a more amorphous structure than SiO_2_ film. This result correlates with the fact that the SiO_x_ film is less dense and has a lower refractive index [44]. In fact, Figure 6b and Figure 8a demonstrate that the variation of film density and refractive index agrees with this result perfectly. Furthermore, the decrease of *E_d_* from SiO_2_ to SiO_x_ sample is also supposed to relate to the effective number of valence electrons, the variation of Si-O and Si-Si bonds, as shown in Figure 4.

Moreover, the long-wavelength limit of the refractive index, n(0), is given by [44]: n^2^(0) = 1+ *E_d_*/*E*_0_. Then n(0) is estimated to be 1.52, 1.49, and 1.39 for SiO_2_, SiO_1.8_, and SiO_1.6_, respectively, as seen in Table 1. For comparison, the refractive index at 632.8 nm obtained by SE analysis are also listed in Table 1. It is easy to find that the value of n(0) is very close to the refractive index at 632.8 nm, except for a little smaller. The excellent agreement of these results improved the correctness of the model.

### 3.3. Photoluminescence Properties of SiO_1.6_/SiO_2_ Super-Lattice

To exam the luminescence property of the Si rich SiO_x_ film prepared in this work, SiO_x_/SiO_2_ super-lattice with x = 1.6 was fabricated. The super-lattice consisted of 22 SiO_1.6_/SiO_2_ bilayers, and the nominal thickness of SiO_1.6_ layer was ~1.5 nm, while the SiO_2_ layer was fixed at ~3 nm, so the total film thickness was about 100 nm. In order to form the Si-NCs in the SiO_2_ matrix, thermal annealing was performed in a rapid thermal annealing furnace under nitrogen atmosphere with ramp rates of about 25 °C/s for phase separation and crystallization. In this work, the sample was annealed at 1000 and 1100 °C for 10 s.

**Figure 10:**
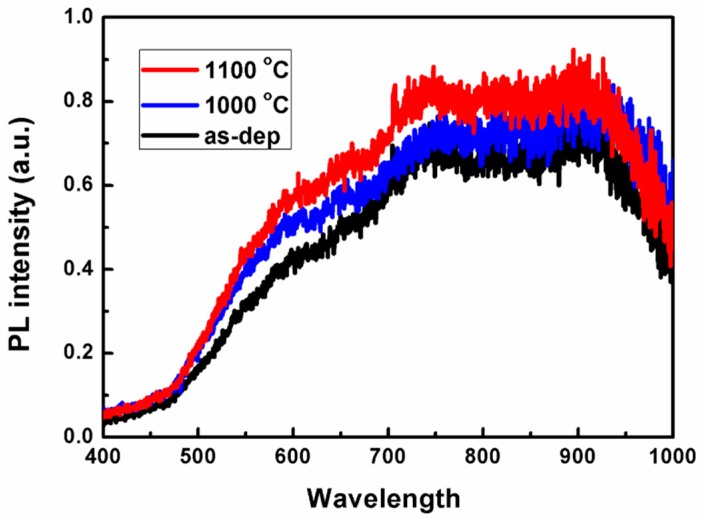
Photoluminescence spectra of the as-deposited and annealed SiO_1.6_/SiO_2_ superlattice.

Figure 10 compares the room-temperature PL spectra of the SiO_1.6_/SiO_2_ super-lattice before and after RTP annealing with different temperature. All of the spectra show the similar luminescence profiles, and the PL maximum position does not show any notable features with annealing temperatures, except for an increase in the PL intensity. In detail, three peaks all have a broader profile ranging from 550 nm to 1000 nm, and two small peaks presents around 750 nm and 920 nm. It seems that the spectra is superimposed by some small peaks, for example, three sub-peaks with gaussian shape located at 600, 750, and 920 nm could form a spectra like this. This result presents a familiar PL spectra for Si-NCs in SiO_x_/SiO_2_ super-lattice deposited by other techniques in previous reports [11,45,46,47,48]. The weak and inapparent luminescence properties of the super-lattice implied that there was not enough Si-NCs formed in SiO_2_ matrix. On one hand, it is supposed that the annealing temperature and time are not sufficient to form Si-NCs compared with the conditions in the previous report [49]. On the other hand, the SiO_1.6_ layer was not Si-rich enough to form considerable Si-NCs when high temperature annealing. Specifically, the high temperature annealing of such initially amorphous SiO_x_ films results in phase separation described by [9],

(2) $${\mathrm{SiO}}_{x}\to \frac{x}{2}{\mathrm{SiO}}_{2}+(1-\frac{x}{2})\mathrm{Si}$$

in SiO_x_ clusters. The phase separation of the SiO_x_ automatically ensures that the nucleated Si nanocrystals/nanoclusters are separated from each other by a SiO_2_ shell. Based on the theory above, it is just 20% of SiO_x_ cluster turned to Si nanocrystals when x = 1.6. Also, as reported in previous articles [50], an increase in the stoichiometry parameter x to 1.63 in SiO_x_/SiO_2_ matrix resulted in a drastically reduced density of nanocrystals correlated with a strong decrease in PL intensity. Based on Equation (2), the thickness of the oxide between the Si nanoclusters depends on the stoichiometry of the SiO_x_ as well. The crystallization of SiO_x_ film resulted in randomly distributed nanocrystals with an average size that depends on the original stoichiometry. So both the thickness of SiO_x_ and SiO_2_ layer also play a role in this property, which should be systematically studying in further research.

## 4. Conclusions

In this study, the SiO_x_ thin film with different stoichiometry was successfully deposited with TDMAS and O_2_ plasma by using PEALD. In order to realize this target, the deposition parameter was firstly studied, and the effect of deposition temperature, plasma pulse time, and gas flow on growth rate was obtained. Then, SiO_x_ thin films with different stoichiometry were fabricated by optimizing the precursor pulse time and O_2_ gas flow. Meanwhile, the micro-structure, chemical, and optical properties were systematically investigated by AFM, TEM, XRR, SE, and XPS measurements. It was found that the mass density, refractive index, and optical energy band-gap of SiO_x_ film are all lower than SiO_2_ film. Furthermore, SiO_1.6_/SiO_2_ superlattice with 150 nm thickness was fabricated based on the deposition parameters obtained above. The photoluminescence test shows that the SiO_1.6_/SiO_2_ super-lattice presents a weak and indistinctive peak before and after high temperature annealing, implying that the SiO_1.6_ film is still not Si rich enough. Further research is worth exploring to obtain the SiO_x_ film with a smaller x value by optimizing PEALD parameters.

## Figures and Tables

**Table 1:** Characteristics of the studied samples ^a^.

Sample	*E*_0_/*E_d_*	(*E*_0_*E_d_*)^−1^	*E*_0_	*E_d_*	*E_g_*	*E_g_* (by O 1s)	n(0)	n/(632.8 nm)
S1 (SiO_2_)	0.757	0.0048	12.52	16.54	8.3	8.0	1.52	1.54
S2 (SiO_1.8_)	0.821	0.0074	10.53	12.83	7.0	7.5	1.49	1.51
S3 (SiO_1.6_)	1.062	0.0099	10.37	9.75	6.9	7.3	1.39	1.42

^a^ Single oscillator and dispersion energies *E*_0_ and *E_d_*, respectively, the optical energy band-gap, *E_g_*, simulated by *E*_0_ and by O 1s peak, refractive index in the long-wavelength limit n(0), and the refractive index n at 632.8 nm from Figure 8a.
